# Mutations in Glucan, Water Dikinase Affect Starch Degradation and Gametophore Development in the Moss *Physcomitrella patens*

**DOI:** 10.1038/s41598-019-51632-9

**Published:** 2019-10-22

**Authors:** Ntombizanele T. Mdodana, Jonathan F. Jewell, Ethel E. Phiri, Marthinus L. Smith, Kenneth Oberlander, Saire Mahmoodi, Jens Kossmann, James R. Lloyd

**Affiliations:** 1 Department of Genetics, Institute for Plant Biotechnology, University of Stellenbosch, Stellenbosch, South Africa; 2 Schweickerdt Herbarium, Department of Plant and Soil Sciences, University of Pretoria, Private Bag X20, Hatfield, 0028 South Africa

**Keywords:** Carbohydrates, Plant physiology

## Abstract

The role of starch degradation in non-vascular plants is poorly understood. To expand our knowledge of this area, we have studied this process in *Physcomitrella patens*. This has been achieved through examination of the step known to initiate starch degradation in angiosperms, glucan phosphorylation, catalysed by glucan, water dikinase (GWD) enzymes. Phylogenetic analysis indicates that GWD isoforms can be divided into two clades, one of which contains GWD1/GWD2 and the other GWD3 isoforms. These clades split at a very early stage within plant evolution, as distinct sequences that cluster within each were identified in all major plant lineages. Of the five genes we identified within the *Physcomitrella* genome that encode GWD-like enzymes, two group within the GWD1/GWD2 clade and the others within the GWD3 clade. Proteins encoded by both loci in the GWD1/GWD2 clade, named PpGWDa and PpGWDb, are localised in plastids. Mutations of either *PpGWDa* or *PpGWDb* reduce starch phosphate abundance, however, a mutation at the *PpGWDa* locus had a much greater influence than one at *PpGWDb*. Only mutations affecting PpGWDa inhibited starch degradation. Mutants lacking this enzyme also failed to develop gametophores, a phenotype that could be chemically complemented using glucose supplementation within the growth medium.

## Introduction

The bryophyte *Physcomitrella patens* is a species that has many advantages for use in the study of plant molecular physiology. Its genome has been sequenced^1^, it is transformable, knockout mutations are easily generated through homologous recombination, and its generally haploid lifestyle means that mutations can be studied directly in the M_1_ generation^2^. *Physcomitrella* and tracheophytes diverged approximately 450 million years ago^1^, after which vascular plants developed an increased ability to survive without proximate water sources. During this time, their metabolic pathways will have changed in a way that would advantage vascular plants in the new ecological niches they encountered. The use of this plant to study metabolism allows for the analysis of how biochemical pathways have altered in different plant species since this divergence^3^.

We have decided to examine the pathway of starch metabolism in *P. patens*. This is because the presence of starch has been demonstrated to be important for plant growth and development in vascular plants^4,5^, and we wish to examine if the same holds true in this non-vascular species. When grown on artificial media, *P. patens* spores or explant material develop initially into thread like protonemal tissue comprising two distinct cell types, chloronema and caulonema. Caulonemal cells contain fewer chloroplasts than chloronemal cells, and can also form buds which develop into leafy shoots known as gametophores. Many factors are known to affect gametophore development including alterations in cell wall^6^, phytohormone synthesis^7,8^, light perception^9^ and several regulatory genetic elements^10–12^. Alterations in carbon metabolism and sensing also alter colony growth and development, alongside influencing starch accumulation^13–15^. Little is known about the role of starch in *Physcomitrella*, although it is present^14,16^ and its degradation has been implicated in freezing tolerance^16^.

In this study we examine the step that has been shown to initiate starch catabolism in angiosperms, starch phosphorylation^17,18^. Much, if not all of the pathway of leaf starch degradation has been recently elucidated through studies in a number of species, primarily *Arabidopsis*, and involves several enzymatic reactions^5,19^. The initial steps occur within chloroplasts where starch is phosphorylated by glucan, water dikinase (GWD) isoforms^20–28^, that solubilize the surface of the granule, allowing access to α-amylase, β-amylase, isoamylase and β-limit dextrinase^29–32^. Degradation by these enzymes releases soluble phosphorylated malto-oligosaccharides into the stroma. The phosphate from these is removed by two phosphoglucan phosphatases^33–35^, before they can be further degraded to maltose and glucose through the actions of ∝-, β- and isoamylases^29–31^ alongside disproportionating enzyme 1^36–38^. Maltose and glucose are exported from the plastid into the cytosol by two transporters^39–41^ where the maltose is further mobilized by disproportionating enzyme 2^37,38,42,43^.

To help understand the role of starch degradation in *P. patens* we decided to mutate some of the starch phosphorylating enzymes as they catalyse the first step in starch degradation. Their importance is, therefore, likely to be conserved between vascular and non-vascular plants. Three of these have been identified in angiosperms: GWD1 is localized to the plastid and phosphorylates glucose residues within amylopectin at the 6-position^20,24^. Mutations in this gene lead to starch without covalently bound phosphate and a large decrease in leaf starch degradation^25,26,44–47^. GWD3 is also plastidial and phosphorylates glucose residues within starch at the 3-position^20^. It is often named the phosphoglucan, water dikinase (PWD) as it can only phosphorylate starch that has already been acted upon by GWD1. Mutations eliminating GWD3/PWD lead to starch without phosphate bound at the 3-position and a mild repression of leaf starch degradation^27,28^. GWD2 is present in the cytosol and the gene encoding it is expressed mainly in sieve elements^48^. Although mutations eliminating it do not affect starch turnover in photosynthetic tissue, they affect plant growth^49^.

In this study we report on mutations in two GWD1 isoforms, and demonstrate that this leads to the synthesis of starch containing reduced glucose 6-phosphate. Mutations in one of the isoforms lead to increased starch accumulation, and to colonies that do not produce gametophores.

## Results

### The *Physcomitrella patens* genome contains multiple GWD isoforms

We examined the presence of sequences encoding GWD-like enzymes within the *Physcomitrella* genome through a tBLASTn search using the *Arabidopsis* GWD1 (NCBI accession NM_001331926.1) amino acid sequence at Phytozome (https://phytozome.jgi.doe.gov/pz/portal.html#!search?show=BLAST). This identified five loci on chromosomes 3 (Pp3c3_11200), 8 (Pp3c8_6536), 14 (Pp3c14_19150), 17 (Pp3c17_18900) and 18 (Pp3c18_14870) encoding putative GWD isoforms. BLASTP searches of the *A. thaliana* genome using the predicted amino acid sequence encoded at each loci indicated that the genes on chromosomes 3 and 8 were most similar to AtGWD1, while the other three were most similar to AtGWD3. We decided to name the two genes that appear to encode GWD1 or GWD2 isoforms as *PpGWDa* (Pp3c8_6536) and *PpGWDb* (Pp3c3_11200), while we named the other three loci *PpGWDc* (Pp3c17_18900), *PpGWDd* (Pp3c14_19150) and *PpGWD*e (Pp3c18_14570).

**Figure 1:**
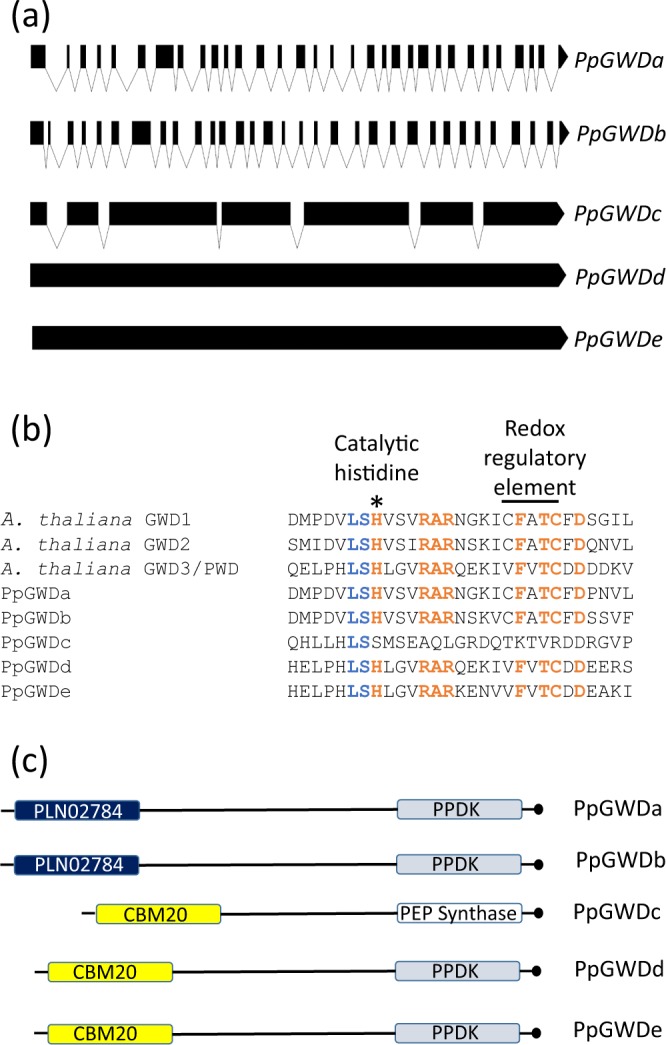
Analysis of putative GWD encoding sequences from *Physcomitrella*. (**a**) Predicted exon-intron structure of the five identified loci. (**b**) Amino acid sequence within the active sites of the predicted proteins in comparison with those from *Arabidopsis*. (**c**) Domain structure within the PpGWD proteins. PLN02784, CBM20, Pyruvate phosphate dikinase (PPDK) and PEP synthase domains are shown at the approximate sites that they are found within the polypeptides. The length of the figures represent the relative number of amino acids (aa) present in PpGWDa (1420aa), PpGWDb (1415aa), PpGWDc (989aa), PpGWDd (1170aa) and PpGWDe (1148aa). Black circles denote the C-termini.

The coding sequences for two of these (*PpGWDa* & *PpGWDb*) are predicted to contain 31 introns, one (*PpGWDc*) contains 6 introns, while the final two (*PpGWDd* & *PpGWDe*) are intronless (Fig. 1a). Four of the five loci (*PpGWDa, PPGWDb, PpGWDd* & *PpGWDe*) encode proteins containing the histidine known to be involved in transfer of phosphate during the dikinase reaction^50^, while the *PpGWD*c locus contains a deletion that eliminates this residue (Fig. 1b). PpGWDa and PpGWDb are approximately 1420 amino acids (aa) in length, PpGWDc is 989 aa and the other two approximately 1160 aa. Amino acid sequences encoded at both *PpGWDa* and *PpGWDb* loci contain the CFATC motif thought to be involved in redox regulation^51^, while in PpGWDd and PpGWDe that motif is altered to VFVTC. The deletion present within *PpGWDc*, which eliminates the catalytic histidine, also removes the VFVTC containing region (Fig. 1b). All polypeptides encoded by these loci contain C-terminal dikinase domains, similar to those from either pyruvate phosphate dikinase or PEP synthase. PpGWDa and PpGWDb contain N-terminal PLN02784 carbohydrate-binding α-amylase domains, whilst the related CBM20 domain is present at the N-termini of PpGWDc, PpGWDd and PpGWDe (Fig. 1c).

**Figure 2:**
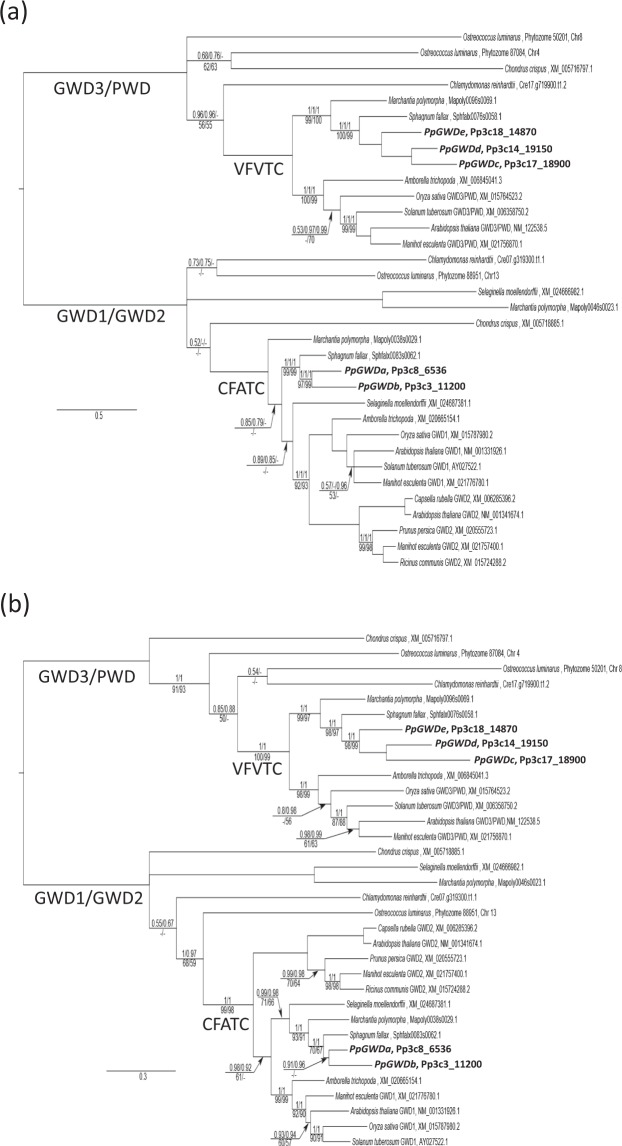
Phylogeny of GWD sequences from various plant species. (**a**) 50% majority rule consensus tree from partitioned MrBayes analysis of the full DNA data set of Archaeplastid GWD/PWD sequences, showing phylogenetic placement of determined *P. patens* (bold) paralogs. The three numbers listed above branches are Bayesian posterior probabilities (PP) for the full, partitioned analysis, PP for a partitioned analysis with a region of uncertain homology excluded, and PP for the recoded RY analysis. Numbers under each branch are RAxML likelihood bootstrap support (BS) for the full, partitioned analysis and BS for a full, partitioned analysis with a region of uncertain homology excluded. Dashes indicate support for a branch <50%, or where the branch was not present in a particular analysis. Un-numbered branches received maximal support in all analyses. The scale bar is in substitutions per site. (**b**) 50% majority rule consensus tree from partitioned MrBayes analysis of the full AA data set of Archaeplastid GWD/PWD sequences, showing phylogenetic placement of determined *P. patens* (bold) paralogs. The two numbers listed above branches are Bayesian posterior probabilities (PP) for the full analysis, and PP for analysis with a region of uncertain homology excluded. Numbers under each branch are RAxML likelihood bootstrap support (BS) for the full analysis and BS for analysis with a region of uncertain homology excluded. Dashes indicate support for a branch <50%, or where the branch was not present in a particular analysis. Unnumbered branches received maximal support in all analyses. The scale bar is in substitutions per site. In (**a**,**b**) the branches subtending two land plant clades characterized by specific motifs are labelled with the corresponding amino acid sequence (VFVTC and CFATC). Sequences are identified either by Phytozome AGI codes or NCBI accession numbers.

A recent phylogeny of GWD sequences has been published^47^, but this only included three of the five *Physcomitrella* sequences, and none from other non-vascular plants. To examine the relationships of the five sequences identified from the *Physcomitrella* genome with other GWD genes and proteins, we have produced new phylogenetic trees including all *Physcomitrella* sequences as well as others from a number of red and green algae, non-vascular and vascular plants. Analysis was performed using both nucleotide (DNA; Fig. 2a) and amino acid (AA; Fig. 2b) data in both a maximum likelihood (RAxML) and Bayesian (MrBayes) context.

All MrBayes runs reached stationarity (all Potential Scale Reduction Factors between 0.99 and 1.01) and achieved adequate sample sizes (minimum ESS across combined runs >400). MrBayes AA runs overwhelmingly (posterior probability = 1) chose the WAG model^52^ as the best-fitting empirical model, which was used for RAxML AA analyses. When rooted on the longest internal branch, the tree consisted of two major clades, one containing GWD3/PWD sequences, and the other containing all GWD1 and GWD2 sequences. The five *Physcomitrella* sequences grouped in two distantly-positioned portions of the tree. The first position, retrieved in all analyses with near-maximal support, contained the three intronless/intron-poor sequences as each other’s closest relatives, with *Sphagnum fallax* (Sphfal0076s0058.1) and *Marchantia polymorpha* (Mapoly0096s0069.1) as successive sisters in all analyses with maximal or near-maximal support. All five bryophyte sequences grouped in a land plant clade with maximal support, and these land plant sequences also uniquely shared the VFVTC sequence. The second position contained the two intron-rich sequences as sister to *Sphagnum fallax* (Sphfal0083s0062.1), with strong support (DNA) and moderate to strong support (AA) in all analyses. DNA- and AA-based analyses differed on the closest relatives of this moss clade, with DNA analyses equivocal and AA analyses supporting *Marchantia polymorpha* (Mapoly0038s0029.1) and *Selaginella moellendorffii* (XM_024687381.1) as successive sisters, with moderate to strong support. These sequences formed part of a strongly supported land plant clade that shared the CFATC redox regulation motif (or minor variants thereof). In addition to the bryophyte/*Selaginella* sequences, this land plant clade included a clade containing all characterized GWD1 accessions, and a last clade containing all characterized GWD2 accessions, but relationships among these differed.

### Both GWDa and GWDb localise to the plastid

**Figure 3:**
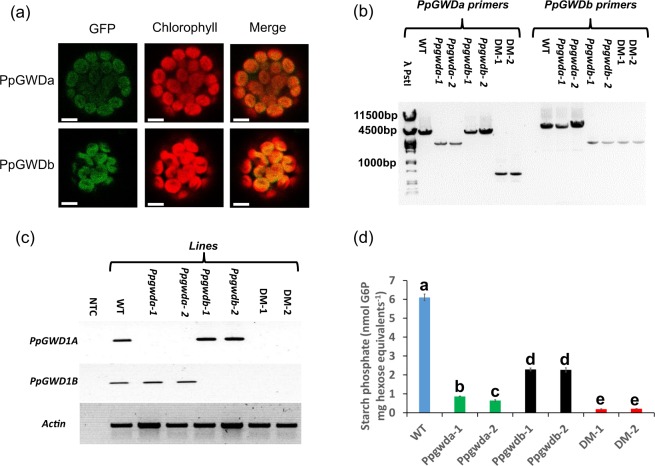
Examination of GWD in *P. patens*. (**a**) Protoplasts expressing PpGWDa or PpGWDb fused to GFP. Scale bar is 5 µm. (**b**) PCR analysis from gDNA demonstrating the presence of mutant alleles in *Ppgwda*, *Ppgwdb* or double mutant (DM) lines. Amplicons were separated on a 1% (w/v) agarose gel. λ-PstI represent λ phage DNA digested with PstI. (**c**) Semi-quantitative RT-PCR analysis of *PpGWD1a*, *PpGWD1b* or *Actin* expression in the wild-type (WT), *Ppgwd1a*, *Ppgwd1b* and DM experimental lines. NTC designates the no template control. Original gels are shown in Supplementary Fig. 2. (**d**) Glucose 6-phosphate amounts in starch from the wild-type and mutant lines. Data represent means of three independent digestions of pooled starch samples ± SEM. Letters represent groups with similar means at the 5% significance level as determined using the Bonferroni-Holm *post hoc* test following a one-way analysis of variance. Equal variance was determined using Levene’s test.

We examined the presence of transit peptides in all five genes using the ChloroP server. PpGWDa, b and e were predicted to target to plastids, while PpGWDc and d were predicted not to contain transit peptides. To confirm the localization of the GWD1 like isoforms we produced constructs where cDNA of either gene was fused in frame with GFP at the C-terminus. These were transformed into *P. patens* protoplasts and transiently expressing samples imaged by confocal microscopy. In both cases the GFP signal coincided exactly with that of chlorophyll, demonstrating that both proteins are plastidial (Fig. 3a).

### Mutations in both genes affect starch phosphorylation

We used homologous recombination to manufacture mutants. The constructs were designed to replace DNA encoding the known dikinase domain from either gene (Fig. 2a and Supplementary Fig. 1), which includes the catalytically essential histidine residue^50^, with a disruption cassette flanked with *loxP* sites. For each gene we recovered two plants that grew on hygromycin following transformation with gene disruption cassettes, and named these *Ppgwda-*1 or -2 and *Ppgwdb*-1 or -2. We also removed the disruption cassettes from both lines containing inserts at the *PpGWDa* locus using cre recombinase and, in these lines, mutated the *PpGWDb* locus to produce two double mutant (DM-1 or -2) lines.

To confirm that the lines we created were mutated, PCR analysis was performed using gDNA template and two sets of primers (Fig. 3b). One primer set, which binds either side of the homologous recombination sites, was used to detect the presence of either wild type alleles (amplicon sizes of 4500 bp for *PpGWDa* and 6000 bp for *PpGWDb*) or deletions within the *PpGWDa* locus in the DM (amplicon size approximately 700 bp). This demonstrated that wild type *PpGWDa* alleles were present in the *Ppgwdb* mutants and wild type *PpGWDb* alleles were present in *Ppgwda* mutants. As expected the PpGWDa amplicon in the DM was approximately 700 bp due to the elimination of the resistance cassette and part of the gene encoding the active site. A second primer set was used where one primer binds upstream of the insertion and another within the disruption cassette. This demonstrated the presence of the cassette within the *PpGWDb* locus in both single and double mutant lines (Fig. 3b). We attempted to examine protein amounts using a GWD antibody that was raised against the potato protein^25^, but it did not recognize any *Physcomitrella* GWD polypeptide. Therefore, we examined gene expression using semi quantitative RT-PCR, and showed that RNA transcribed from either *PpGWDa* or *PpGWDb* loci was abolished whenever the appropriate gene disruption cassette was present (Fig. 3c and Supplementary Fig. 2).

As GWD isoforms are known to phosphorylate starch in vascular plants, we examined the amounts of covalently bound starch phosphate in both the single and double mutants (Fig. 3d). There was a significant decrease in all the mutant lines. Starch from *Ppgwda* mutant lines contained approximately one seventh of the glucose 6-phosphate of the control, while lines containing an insert within the *PpGWDb* locus contained approximately one third. Glucose 6-phosphate was reduced below that of the *Ppgwda* lines in the double mutants.

### Mutations in *PpGWDa a*ffect starch degradation

**Figure 4:**
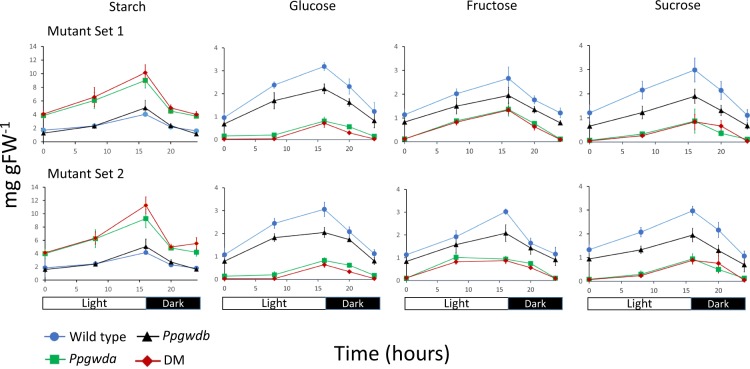
Starch and soluble sugar amounts in colonies from the WT and mutant lines. Plants were grown on BCD medium for 5 weeks under a 16 h/8 h day/night regime. Tissue was sampled at five time points over a 24 hours period and starch, glucose, fructose and sucrose determined. Data represent means of at least 5 colonies. Error bars are SEM and, if not visible, are within the symbol.

To examine starch degradation in the mutant lines, we grew colonies for 8 weeks on artificial medium before determining starch and soluble sugars over a diurnal cycle from entire colonies (Fig. 4). Starch contents increased during the light period and decreased during the dark. The WT and *Ppgwdb* mutants accumulated similar amounts of starch at all time points, as did the *Ppgwda* and DM mutants. Starch was significantly lower (p ≤ 0.05) in the WT and *Ppgwdb* lines in comparison with *Ppgwda* and DM mutants at almost all time points. The one exception was at 0 hours in the second set of mutants when the starch content in the WT and the *Ppgwda* mutant were invariable. Conversely, soluble sugars were reduced in the *Ppgwda* and DM mutant lines compared with the WT and *Ppgwdb* mutant. Within both mutant sets glucose was significantly (p ≤ 0.05) reduced in the *Ppgwda* and DM lines compared with the others at the 0, 8, 16 and 20 hour time points. Similarly fructose was significantly (p ≤ 0.05) reduced at 20 and 24 hours and sucrose at 0, 8, and 20 hours.

### *Ppgwda* and DM plants demonstrate altered colony morphology

**Figure 5:**
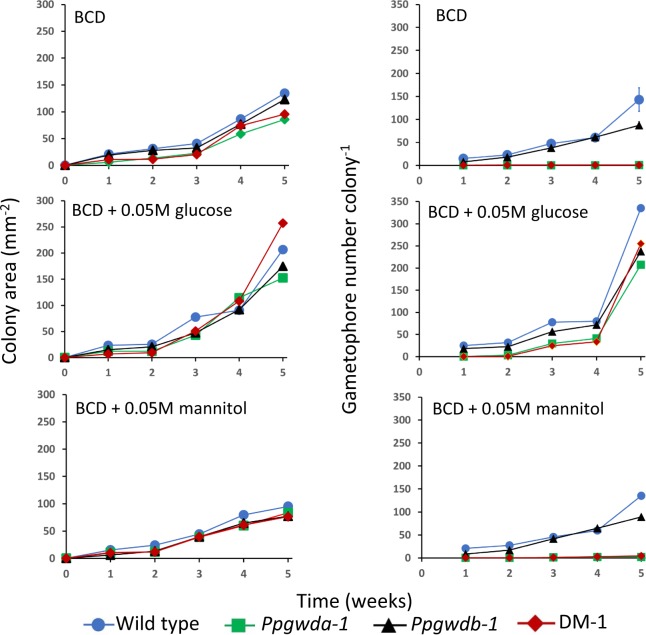
Growth and gametophore number in the first set of mutant lines. Colonies were established on BCD media, BCD + 0.05 M glucose and BCD + 0.05 M mannitol and allowed to grow for 5 weeks. Data represent means of at least 3 colonies. Error bars are SEM and, if not visible, are within the symbol.

There were no consistent significant differences in growth between the lines when grown on BCD medium, however, we noted alterations in colony morphology with the WT and *Ppgwdb* lines developing gametophores, while the *Ppgwda* and DM lines did not. Data from the first mutant set are presented in Figs 4 and 5, while data from the second set are presented in Supplementary Figs 3 and 4. Colonies from the wild type and *Ppgwdb* mutants contained significantly (p < 0.05) more gametophores compared with *Ppgwda* and DM lines at every time point, when grown on BCD media or BCD media supplemented with mannitol. Under these growth conditions Ppgwdb mutants also accumulated significantly (p < 0.05) fewer gametophores than the wild-type at week 5. When the plants were grown on media containing glucose, all lines grew faster and all produced significant numbers of gametophores (Fig. 5 and Supplementary Fig. 3). The *Ppgwda* and DM lines still contained significantly fewer gametophores (p < 0.05) than the WT at weeks 1 and 5, but at weeks 2, 3 and 4 they contained similar numbers (Fig. 5 and Supplementary Fig. 3).

## Discussion

Starch degradation is important for the normal growth and development of some angiosperms^5^, but knowledge about its role in these processes in non-vascular plants is lacking. Some work has been initiated in *Chlamydomonas* where a forward genetic approach has identified mutants affecting starch catabolism^53^, but to date only plants mutated in the plastidial maltose transporter have been characterized^54,55^. To broaden our knowledge of this process in non-vascular plants, we have examined the role of GWD1 like enzymes in *Physcomitrella patens* through production of targeted mutants.

Analysis of the *Physcomitrella* genome identified five genes encoding proteins with significant similarity to GWD1 from *Arabidopsis* (Fig. 1). Phylogenetic analyses (Fig. 2a,b) demonstrated the presence of two clades, each containing red algal, green algal and land plant representatives in an approximate recapitulation of recognized Archaeplastid phylogeny. This indicates that the duplication leading to GWD3/PWD-type genes and GWD1/GWD2-type genes was present in the last common Archaeplastid ancestor; yet we could find no clear homologs to any GWD-type gene in *Gloeomargarita lithophora*, the recently identified cyanobacterium thought to be sister to the plastid endosymbiont^56^. Subsequent duplications led to different isoforms of *Ostreococcus*-type GWD3/PWD genes, an uncharacterized GWD1/GWD2-type lineage shared by *Selaginella* and *Marchantia*, and the separation of GWD1 and GWD2 clades somewhere between the evolution of tracheophytes and angiosperms.

The differing topologies in the DNA/AA phylogenies suggest two potential evolutionary scenarios: the DNA-based trees (Fig. 2a) indicates a duplication event leading to the formation of GWD2 isoforms after the divergence of bryophytes and tracheophytes i.e. acquisition of GWD2 function and localization from a GWD1-type ancestor. Alternatively, the AA trees (Fig. 2b) suggest a much deeper separation of GWD1/GWD2 lineages, at or near the origin of land plants, but with GWD2 genes either deleted from or undetected in all non-angiosperm land plants, and ancestral function uncertain. Our analysis indicates that there are two *P. patens* genes (*PpGWDa* and *PpGWDb*) encoding either GWD1 and/or GWD2 isoforms. Like AtGWD1^26^, but unlike AtGWD2^48^, both encode polypeptides that are targeted to plastids (Fig. 3a). This may indicate that the interpretation from the DNA tree is correct, but similar analysis of GWD1/GWD2 like isoforms in green algae, other non-vascular plants, seedless vascular plants and gymnosperms will be needed to confirm this. The other three genes are most likely GWD3/PWD isoforms as they group in that clade and all contain CBM20 motifs that are found in GWD3/PWD, but not GWD1 isoforms^17^ (Fig. 1c). Interestingly the two *PpGWD1/GWD2* genes contained similar exon intron structures, while *PpGWDd* and *PpGWDe* were intron less (Fig. 1a). All four of these genes encode proteins predicted to contain the known active site, and elements identified in vascular plant GWD1’s as redox regulatory, or variants of them (Fig. 1b)^50,51^. These GWD1/GWD2 and GWD3/PWD gene pairs were most likely formed during the recent genome duplication event that is thought to have occurred in *Physcomitrella* 30–60 million years ago, and which is known to have led to increased numbers of genes involved in metabolic processes^57^. *PpGWDc* contains 7 exons, but also a large deletion. It, therefore, encodes a protein significantly smaller than the others and lacks the catalytically essential histidine^58^ (Fig. 1), meaning that it is unlikely to be active.

To functionally examine the roles of the PpGWD1/PpGWD2 like enzymes, we manufactured mutants in each gene as well as isolating double mutants (DM) lacking both (Fig. 3b,c). The homologous recombination constructs used to produce the mutants were designed to remove parts of the genes encoding the known active site of the protein (Fig. 2b and Supplementary Fig. 1) meaning that any RNA manufactured from the remaining gDNA would encode an inactive polypeptide. The inserts would, however, potentially allow expression of RNA from gDNA upstream of the insert sites, which includes the PLN02784 glucan binding domains. We cannot, therefore, rule out the possibility that protein produced from this could affect starch metabolism in some way. We believe that this is unlikely as point mutations in the *AtGWD1* gene allow production of inactive protein containing the carbohydrate-binding domain, but demonstrate a similar phenotype as knockouts^26^. For both genes we produced two independent sets of single or double mutants. We examined if the mutations reduced starch bound phosphate and found that insertions in either gene reduced the amount of glucose 6-phosphate in starch (Fig. 3d). The reduction was greater when the insertion was present in the *PpGWDa* locus than when *PpGWDb* was mutated. Starch phosphate in the double mutants was, however, lower than either single mutant line. This demonstrates that both genes encode proteins that incorporate phosphate at the 6-position of glucose moieties within the starch polymer *in vivo*, the known biochemical function of GWD1 isoforms, and that PpGWDa plays a greater role in this process than PpGWDb.

Reduction in starch phosphate caused by mutations in GWD1 has been demonstrated to impair starch catabolism in angiosperms^25,26,45,46^. This is believed to be due to the phosphate disrupting double helices between amylopectin chains and allowing access to amylases to initiate starch granule degradation^17,18^. To examine this we grew plants over a diurnal cycle and examined starch and soluble sugar levels (Fig. 4). As in angiosperms starch increased during the light period and decreased when it was dark. Both *Ppgwda* and DM lines contained significantly more starch than the WT or *Ppgwdb* line at almost all time points. Conversely, soluble sugar contents were reduced at most time points in the DM and *Ppgwda* mutants compared with the WT and *Ppgwdb* lines (Fig. 4). This demonstrates that mutations affecting PpGWDa reduce starch degradation in *P. patens* as *Ppgwda* and DM mutant plants develop a starch excess phenotype, but that PpGWDb has little effect on this process. Plants lacking PpGWDa can clearly still degrade starch. Indeed they degraded more starch between time points than either the WT or *Ppgwdb* (Fig. 4), most likely due to the larger amounts they accumulated prior to the start of the experiment. This is similar to other plants lacking GWD where starch is still degraded in leaves over a diurnal cycle, but where a starch excess phenotype develops over time due to small differences in the amount of starch that is mobilised at night^25,26,46^.

The influence of the two enzymes on starch catabolism is in line with their observed effects on starch phosphate amounts, where PpGWDa has a greater influence than PpGWDb (Fig. 3d). However, the relationship between starch phosphate reduction and starch catabolism is not linear as mutations in PpGWDb reduce starch phosphate by more than 50%, but do not affect starch degradation. This indicates both that starch phosphate must be reduced below a threshold level before an effect on starch degradation is observed, and that the starch phosphate is above that threshold in the WT and *Ppgwdb* mutants, but below it in the *Ppgwda* and DM lines. Interestingly a previous study^59^ in *Arabidopsis* demonstrated that GWD amounts had to be reduced below a threshold level before starch degradation was inhibited, although the authors did not examine starch phosphate in that study.

**Figure 6:**
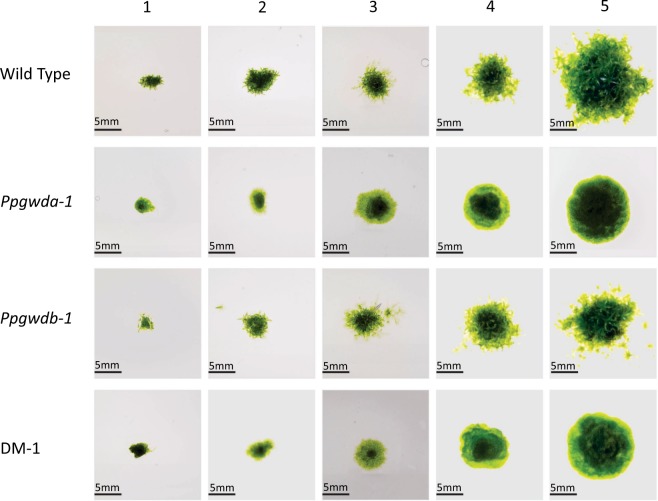
Colony morphology of wild-type and the first set of mutant lines grown on BCD medium for 1, 2, 3, 4 and 5 weeks.

Mutant plants lacking PpGWDa demonstrated an altered appearance when grown on BCD medium, with both *Ppgwda* and DM plants developing almost no gametophores (Figs 5, 6 and Supplementary Figs 3 and 4). We hypothesized that the decrease in starch degradation would reduce soluble sugars within plant cells, and that this could impact both growth and plant development. To examine this, we grew plants on media containing either 0.05 M glucose or 0.05 M mannitol. When placed on glucose-containing medium, all plants grew more quickly and both *Ppgwda* and DM lines developed gametophores, although not to the same extent as wild type or *Ppgwdb* mutants. Neither the increase in growth nor gametophore development occurred when *Ppgwda* or DM lines were grown on mannitol-containing medium, indicating that the reversion of the mutant phenotype is caused by glucose uptake and not the altered osmotic potential. Interestingly mutations in *PpGWDb* also reduced gametophore number to about two thirds of the wild-type strain at week 5. It is not clear why this is the case as we could not identify decreased starch degradation in these lines. It is possible that *PpGWDb* is present at specific growth stages, or in only some cell types, leading to a localized reduction in starch degradation that impacts on gametophore development.

These data demonstrate that starch-derived soluble sugars influence the development of gametophores in *P. patens*. It is possible that decreased soluble sugars inhibit gametophore development directly due to reduction in carbon skeletons, possibly through decreasing substrate supplies to a cellulose synthase which are essential for gametophore development^6^. It is, however, also possible that downstream processes repressing gametophore initiation are affected by the reduced sugar levels. The development both of gametophore forming caulonemal tissue and gametophores is known to be under hormonal control, being influenced by auxins^7^ and cytokinins^8^, as well as a recently identified ancestral gibberrelin^60^. It is unclear if amounts of any of these hormones have been altered to lead to this phenotype, although it is interesting to note that mutations affecting starch degradation in higher plants affects gibberellin synthesis^61^. Our future work will examine alterations in these factors within the *Ppgwd1a* mutant to identify the underlying changes that inhibit gametophore formation.

## Methods

### Analysis of *GWD* genes from *P. patens* and comparison with those from other species

tBLASTn searches of the *Physcomitrella patens* genome were performed using the GWD1 sequence from *Arabidopsis thaliana* (UniProtKB# Q9SAC6) at Phytozome 12.1.6. Genomic sequences of putative GWD encoding genes were downloaded and predicted exon-intron boundaries were visualized at www.wormweb.org/exonintron. The presence of predicted chloroplast transit peptides was examined using ChloroP 1.1^62^ (http://www.cbs.dtu.dk/services/ChloroP/) and conserved domains within predicted polypeptides were identified through searches of the CCD database^63^ at https://www.ncbi.nlm.nih.gov/Structure/cdd/wrpsb.cgi.

DNA sequences encoding GWD polypeptides were obtained from either the NCBI or Phytozome and used to construct a phylogenetic tree. DNA encoding the predicted PPDK domains from *GWD* were translated to amino acid sequence and aligned using Muscle^64^ in MegaX^65^. This was followed by subsequent manual alignment of clearly homoplasious indels, as well as of a region encoding amino acids 214–304 from the start of the alignment, which was highly variable. Phylogenetic analyses were conducted using the CIPRES^66^ implementation of MrBayes 3.2.6^67^ and RAxML v8.2.4^68^, using both the nucleotide (DNA) and amino acid (AA) alignments. All DNA analyses were partitioned by codon position, which yielded vastly improved log likelihood values over naïve analyses (mean Δln likelihood values MrBayes: >1000; Δln likelihood values RAxML: >540). MrBayes partitions were allowed to average over GTR submodels (lset nst = mixed) and assigned a gamma correction for among-site rate variation. All MrBayes analyses were run twice, with 1 million generations and default burnin fractions. All runs were checked for stationarity and adequate estimated sample sizes using MrBayes’ own diagnostics and the program Tracer v1.6–1.7^69^. RAxML DNA partitions were individually assigned the GTRGAMMA model under otherwise default settings, with the bootstopping criterion to determine adequate bootstrap replicates^70^. In addition, because of >35% difference in GC content across sampled sequences, which can bias phylogenetic inference^71^, nucleotide alignments were also transformed to RY-coding (purine-pyrimidine coding) to check for artefacts introduced by base composition heterogeneity and run in MrBayes under the previously mentioned parameters. AA alignments in MrBayes were allowed to average over a set of empirical substitution rate matrices, with a gamma correction applied. Otherwise MrBayes AA analyses were run under the same settings and checked in the same way as the nucleotide results. RAxML AA results used the best-fitting empirical matrix from the MrBayes runs as default, and otherwise were run as for the DNA results. Finally, both DNA and AA matrices were run excluding the highly variable region as defined above, to check whether this region of uncertain homology unduly influenced results. All trees were rooted on the longest internal branch, which was also the branch chosen by midpoint rooting.

### Plant material and growth conditions

*P. patens* ssp*. patens* strain Gransden was a kind gift of Dr. P. Hills (Institute for Plant Biotechnology, Stellenbosch University, South Africa). Standard growth conditions for cultures were 23 ± 2 °C with a light intensity of 50 μmol photons m^−2^ s^−1^ (Osram L 58 V/740, Germany) and a 16/8 hour light/dark regime. Cultures used for starch phosphate analyses were grown on PpNH_4_ medium (1 mM MgSO_4_, 1.85 mM KH_2_PO_4_, 10 mM KCL, 45 mM FeSO_4_, 1 mM CaCl_2_, 9.93 µM H_3_BO_3_, 0.103 µM Na_2_MoO_4_, 0.266 µM CoCl_2_, 0.191 µM ZnSO_4_, 1.97 µM MnCl_2_, 0.169 µM KI, 0.22 µM CuSO_4_, 0.57 µM Al_2_(SO_4_)_3,_ 5 mM diammonium tartrate) supplemented with 1% (w/v) sucrose. For analysis of growth rates and plant morphology, colonies were divided into approximately 2mm^2^ pieces and placed on BCD media (1 mM MgSO_4_, 1.85 mM KH_2_PO_4_, 10 mM KNO_3_, 45 mM FeSO_4_, 1 mM CaCl_2_, 9.93 µM H_3_BO_3_, 0.103 µM Na_2_MoO_4_, 0.266 µM CoCl_2_, 0.191 µM ZnSO_4_, 1.97 µM MnCl_2_, 0.169 µM KI, 0.22 µM CuSO_4_, 0.57 µM Al_2_(SO_4_)_3_) or the same media supplemented with either 0.05 M glucose or 0.05 M mannitol.

### Silencing construct preparation

Genomic DNA was extracted from *P. patens* by the protocol of Edwards *et al*.^72^. Using this as template, amplicons encoding sections of glucan, water dikinase (*PpGWD1a* or *PpGWD1b*) genes were amplified by PCR with gene specific primers (GWDa FWD 5′TTCAGCCAGATAGCGTCGTC3′ and GWDa REV 5′GCCATGCACAAGTCGCTATG3′; GWDb FWD 5′TTGCAGGCGGCTTCTGAACTA3′ and GWDb REV 5′GCTCCCACAAGTGTCTCTCC3′). Amplicons were subsequently cloned into the pJET1.2/blunt vector using a CloneJET PCR Cloning Kit, according to manufacturer’s specifications (Thermo Scientific, Waltham, MA, USA). The plasmid containing *PpGWDa* was digested with SalI and SphI before a loxP-flanked Hygromycin B phosphotransferase (*hph*) resistance cassette was excised from the pMBLH8a^73^ plasmid by digesting with the same enzymes and ligating into the genomic fragment. The plasmid containing the *GWDb* genomic fragment was restricted using StuI and ClaI and the same *hph* resistance cassette was excised from pMBLH8a using EvoRV and ClaI, before being ligated into the *GWDb* genomic DNA. The resulting mutations constructs (PpGWDa-HygR-KO and PpGWDb-HygR-KO) were digested with either NotI (PpGWDa-HygR-KO) or XbaI (PpGWDb-HygR-KO) to produce linear plasmids for transformation.

### Production of single and double mutants

Protoplasts were transformed using PEG mediated transformation and mutant plants recovered following selection^74^. For production of double mutants the disruption cassette in the *Ppgwda* single mutant was removed using Cre recombinase. The DNA sequence encoding Cre-recombinase was amplified by PCR from pMM23^75^ using the following primers: Cre Forward 5′ATGTCCAATTTACTGACCGTAC3′ and Cre Reverse 5′CTAATCGCCATCTTCCAGC3′. Purified amplicon DNA was cloned into the pCR8/GW/TOPO according to the manufacturers (Life Technologies, USA-CA) specifications. The expression cassette from pBinAR-Hyg^76^ was excised using the restriction enzymes EcoRI and HindIII, and ligated into the same sites within pCAMBIA2200 (http://www.cambia.org/daisy/cambia/585). A Gateway reading frame cassette (Gateway Vector Conversion Kit, Thermo Fisher Scientific) was ligated in the SmaI site of the expression cassette polylinker in sense orientation with respect to the 35S promoter. The cre-sequence was transferred into this vector using LR clonase (Life Technologies) as described in the manufacturer’s instructions to obtain pCAMBIA2200-Cre. Protoplasts isolated from *Ppgwda* mutant lines were transformed with pCAMBIA2200-Cre using PEG^74^. Protoplasts were regenerated on BCDAT (BCD medium containing 5 Mm ammonium tartrate) medium lacking antibiotics for two weeks, after which they were transferred to BCDAT containing 50 µg/mL geneticin. After one 1 week, colonies that had survived the selection stage were divided into two, plated on both selective (BCDAT plus Hygromycin-B) and non-selective (BCDAT) media and allowed to grow for another week. Replicates growing only on non-selection medium were screened for loss of *hph* resistance cassette. Plants confirmed as lacking the disruption cassette and containing the deletion within *PpGWD1a* were further mutated using PpGWD1b-HygR-KO using the same method as for the production of single mutants.

### Semi-quantitative reverse-transcriptase PCR

Total RNA was extracted from 2 week old *P. patens* protonemal tissue using Qiagen RNeasy® Plant Mini Kit (Whitehead Scientific, Cape Town, South Africa), according to the manufacturer’s instructions. Synthesis of cDNA was performed with the purified RNA by using the RevertAid™ H Minus First Strand cDNA Synthesis Kit (Thermo Scientific, Waltham, MA, USA). The following primers were used in the PCR: GWD1AsqFwd 5′ACTTGTGCATGGCAGTGTT3′ GWD1AsqRev 5′AACGACGTACAGTTCACCATC3′, GWD1BsqFwd 5′GTGGATCCGTCTTCCAACAT3′ GWD1BsqRev 5′AATATGCTCCCACAAGTGTCTC3′ to examine expression of *PpGWD* genes. The linear amplification range was determined using *P. patens* actin primers^77^ (ActsqFwd 5′AAGGCGAACAGGGAGAAGAT3′; ActsqRev 5′TCCACGAGACGACGTACAAC3′), and 20 cycles was chosen as the optimum number of cycles to use for all semi-quantitative RT-PCR reactions.

### GFP fusions and confocal microscopy

GWDa and GWDb cDNA were amplified by PCR using the following primers: PpGWD1a-GFP forward (5′ATGCAGAGACACGGAGTTCT3′) and reverse (5′TTCAAACGAGACCCCAGATG3′); PpGWD1b-GFP forward (5′ATGAAGAGCTTCAGAGCTCA3′) and reverse (5′TTCAAACAAGACCGCAAATG3′) and cloned into pENTR/D-TOPO (ThermoFischer Scientific). The inserts were transferred to the pMPL1382 GFP vector (http://labs.biology.ucsd.edu/estelle/moss2.html) using LR clonase. The GFP vectors were linearized using SphI and protoplasts were transformed with this DNA as described above. Protoplasts transiently expressing GFP were imaged using a Carl Zeiss LSM780 confocal microscope with an ELRYA S.1 super-resolution platform. GFP signal was detected using a laser excitation of 488 nm and GFP filter detection range of 490–579 nm. Chloroplast auto-fluorescence was detected using a laser excitation of 405 nm and chlorophyll filter detection range of 625–738 nm. Images were analysed with the Zen (black edition, version 2.3) imaging software (Carl Zeiss, Germany).

### Growth and gametophore analysis

Plants grown on BCD medium or BCD supplemented with sugars were imaged and surface area determine by image analysis using ImageJ 1.52a^78^. Gametophore numbers were counted manually.

### Starch, sugar and starch phosphate determination

Starch was measure enzymatically following digestion to glucose. Briefly, 20–50 mg plant material was placed in a microcentrifuge tube and sugars were removed by adding 1 mL of 80% (v/v) ethanol and heating at 95 °C for 1 hour before the ethanol was decanted. This step was repeated twice more. After the final wash, 0.4 mL of 0.2 M KOH was added and the sample heated at 95 °C for one hour before the KOH was neutralized through addition of 70 μL of 1 M acetic acid. At this point 1.5 mL of 100 mM NaAC (pH 5.5) was added alongside 65 U of amyloglucosidase (*Aspergillus niger*, Megazyme) and 60 U of thermostable α-amylase (*Bacillus* sp., Megazyme). This was incubated at 50 °C for 1 hour before 100 μL was mixed with 200 μL of 100 mM Tris-HCl (pH 7.0), 5 mM MgCl_2,_ 1 mM ATP and 1 mM NAD. This was assayed at 340 nm in a microtitreplate reader for increase in absorbance after addition of 1 U of hexokinase (Yeast, Megazyme) and 0.5 U of glucose 6-phosphate dehydrogenase (*Leuconostoc mesenteroides*, Megazyme). Glucose, fructose and sucrose were determined in the ethanol extracts by a previously described method^79^. Starch phosphate was determined by a previously published method^80^ using starch purified^81^ from protonemal tissue.

### Statistical analysis

Data were analysed by one way analysis of variance followed by a Bonferroni-Holm post hoc test using Daniel’s XL Toolbox add-in for Excel^82^, version 7.3.2 (www.xltoolbox.net). Equal variance was demonstrated using Levene’s test^83^.

## Supplementary information

Supplementary Figures
