# Supplementary material for: Mutations in Glucan, Water Dikinase Affect Starch Degradation and Gametophore Development in the Moss Physcomitrella patens
Source: Sci Rep. 2019 Oct 22;9:15114. doi: 10.1038/s41598-019-51632-9 (PMC6805951; doi:10.1038/s41598-019-51632-9)
Supplement: Supplementary file 1 — Supplementary Figures [file 41598_2019_51632_MOESM1_ESM.pdf]

Mutations in Glucan, Water Dikinase Affect Starch Degradation and Gametophore Development in the Moss *Physcomitrella patens.*

Ntombizanele T. Mdodana, Jonathan F. Jewell, Ethel E. Phiri, Marthinus L. Smith, Kenneth Oberlander, Saire Mahmoodi, Jens Kossmann, James R. Lloyd

Supplementary Figure 1. Construct synthesis for mutant production. PCR was used to amplify parts of *PpGWDa* (a) or *PpGWDb* (b) genomic DNA. Arrows indicate approximate primer binding sites within the genes and the H represents the position of the codon encoding the catalytically essential histidine. The amplicons were ligated into pJET1.2/blunt and digested with the restriction sites shown in the gDNA. A resistance cassette containing the cauliflower mosaic virus 35S promoter (*CaMV-35S*), *Klebsiella* hygromycin B phosphotransferase (*hph*) and the cauliflower mosaic virus terminator (*CaMV-ter*) flanked with *loxP* sequences were ligated between those sites.

Supplementary Figure 2. Original DNA gels examining expression of *PpGWD1a*, *PpGWD1b* or *PpActin* via semi-quantitative RT-PCR in the wild-type (WT) or mutant lines. NTC denotes no template control and λPstI is DNA isolated form λ phage digested with PstI and used as a molecular weight marker.

Supplementary Figure 3. Growth and gametophore number in the second set of mutant lines. Colonies were established on BCD media, BCD + 0.05M glucose and BCD + 0.05M mannitol and allowed to grow for 5 weeks. Data represents means of at least 3 colonies. Error bars are SEM and, if not visible, are within the symbol.

Supplementary Figure 4. Colony morphology of wild-type and second set of mutant lines grown on BCD medium for 1, 2, 3, 4 and 5 weeks.

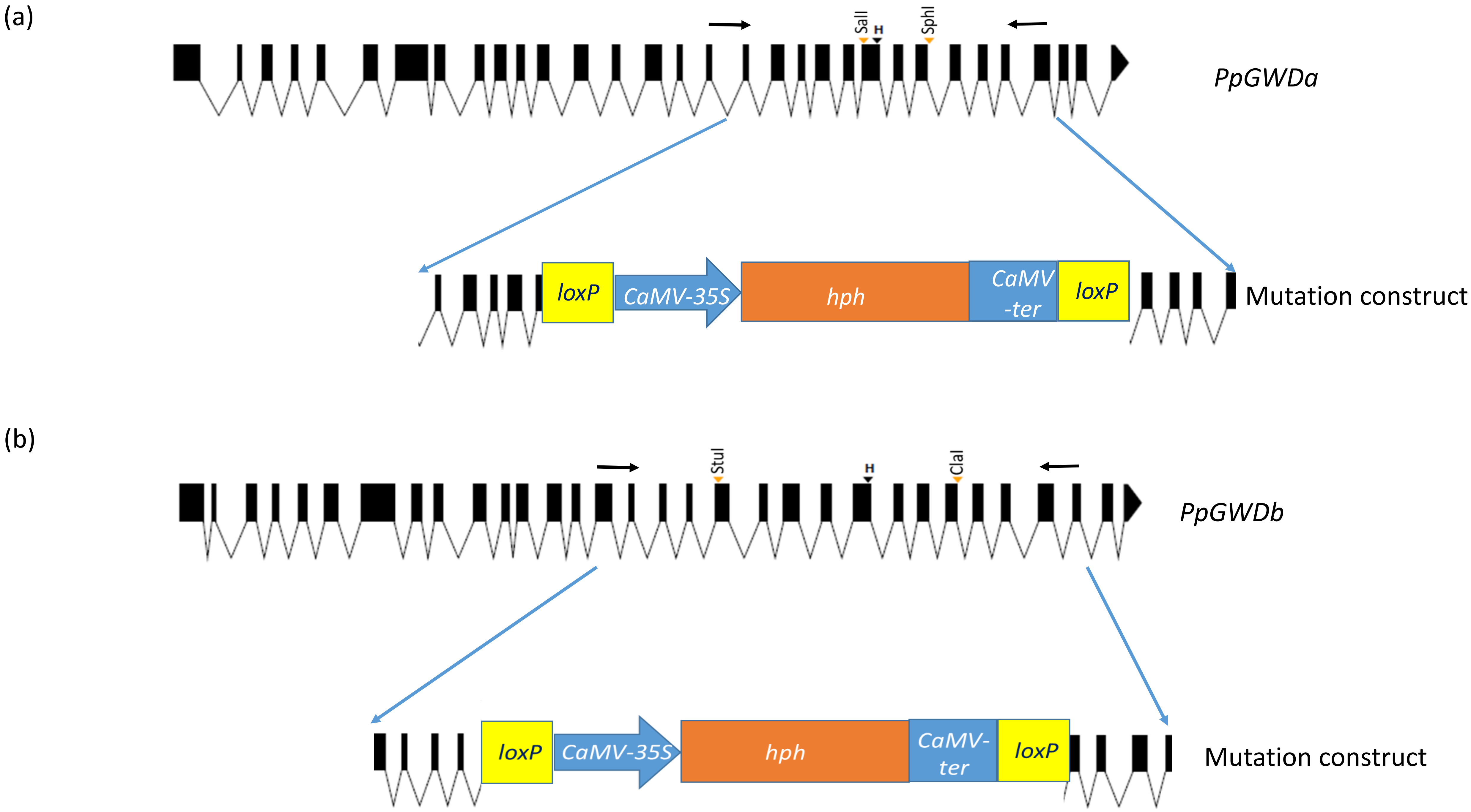

(a)
SalI
H
SphI
PpGWDa
loxP
CaMV-35S
hph
CaMV
-ter
loxP
Mutation construct
(b)
StuI
H
ClaI
PpGWDb
loxP
CaMV-35S
hph
CaMV-
ter
loxP
Mutation construct

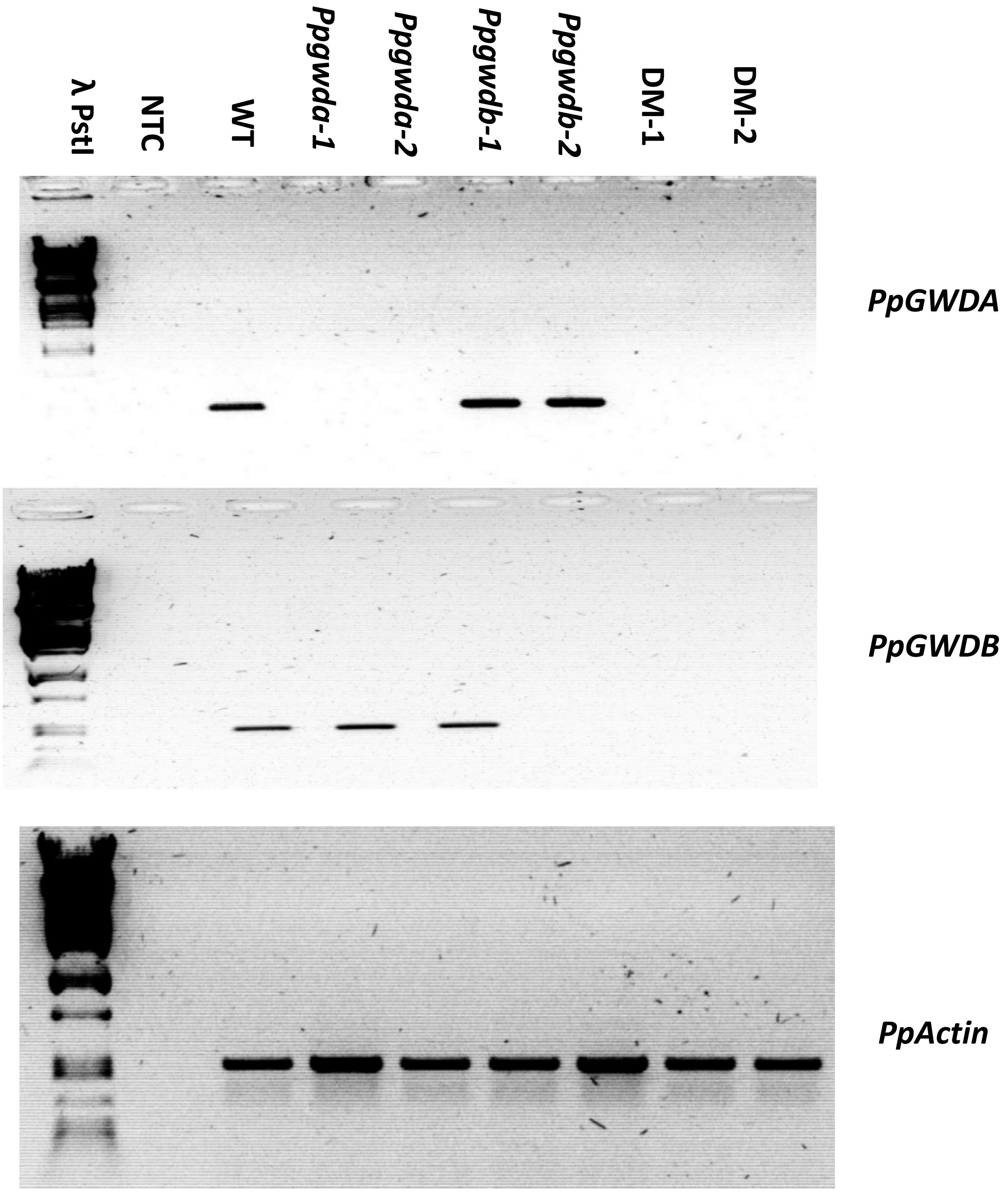
λ PstI
NTC
WT
Ppgwda-1
Ppgwda-2
Ppgwdb-1
Ppgwdb-2
DM-1
DM-2
PpGWDA
PpGWDB
PpActin

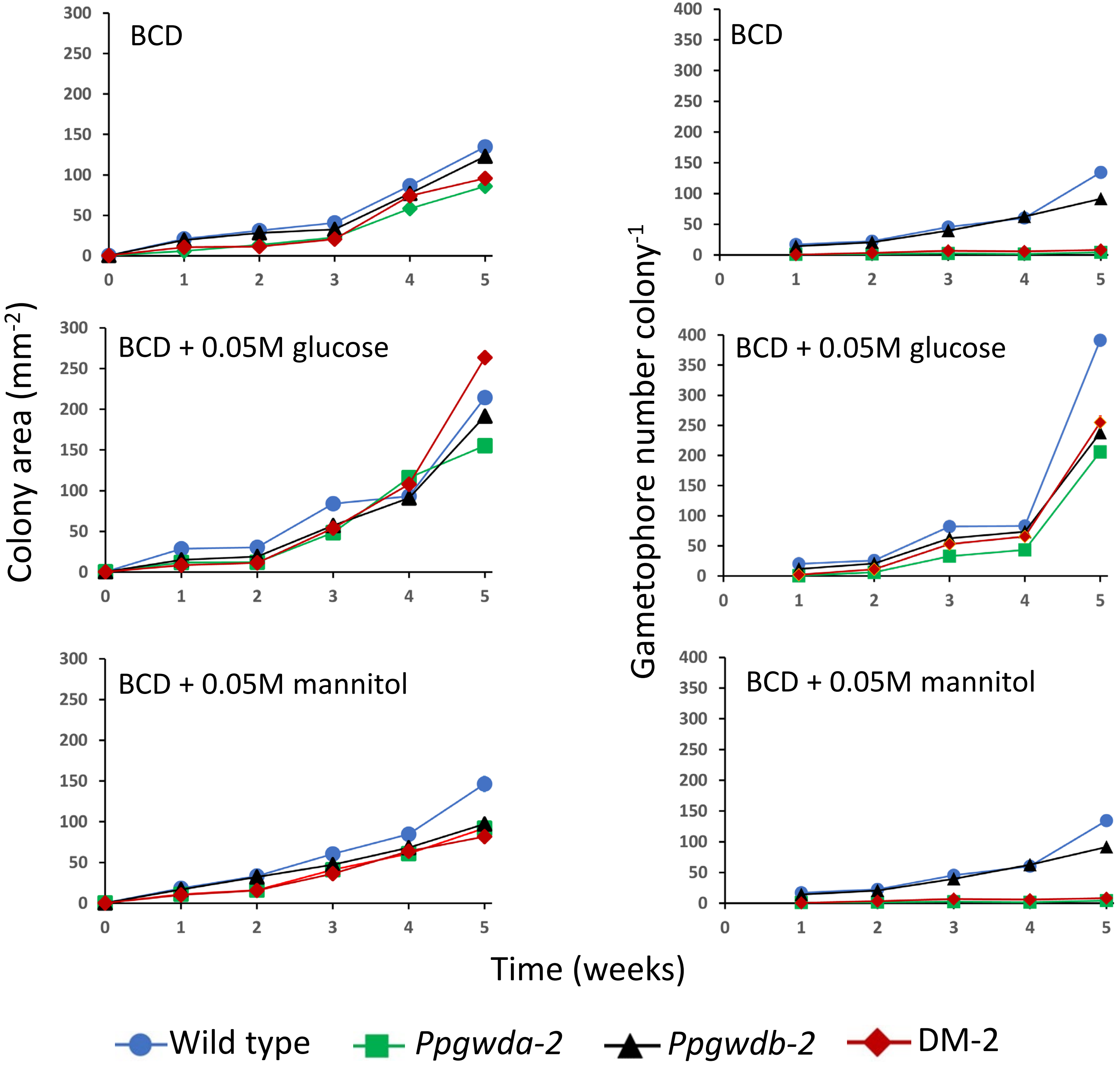

BCD
BCD + 0.05M glucose
BCD + 0.05M mannitol
Colony area (mm^{-2})
Gametophore number colony^{-1}
Time (weeks)
Wild type
Ppgwda-2
Ppgwdb-2
DM-2

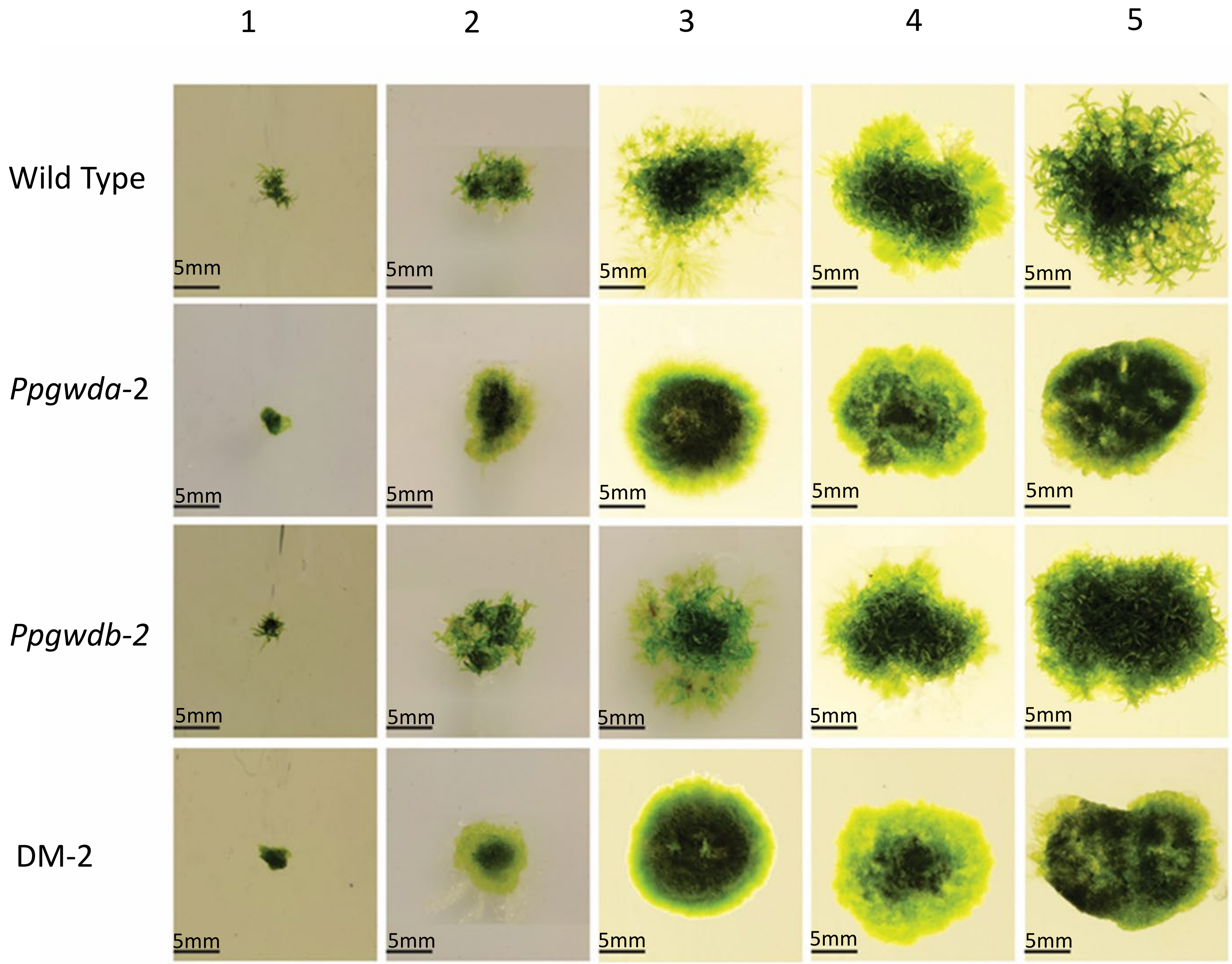
1
2
3
4
5
Wild Type
Ppgwda-2
Ppgwdb-2
DM-2
5mm
